# Genetic effects of *PDGFRB* and *MARCH1* identified in GWAS revealing strong associations with semen production traits in Chinese Holstein bulls

**DOI:** 10.1186/s12863-017-0527-1

**Published:** 2017-07-03

**Authors:** Shuli Liu, Hongwei Yin, Cong Li, Chunhua Qin, Wentao Cai, Mingyue Cao, Shengli Zhang

**Affiliations:** Department of Animal Genetics and Breeding, College of Animal Science and Technology, Key Laboratory of Animal Genetics and Breeding of Ministry of Agriculture, National Engineering Laboratory for Animal Breeding, China Agricultural University, Beijing, 100193 China

**Keywords:** Association analysis, Semen production traits, Candidate genes, Gene expression, Holstein bulls

## Abstract

**Background:**

Using a genome-wide association study strategy, our previous study discovered 19 significant single-nucleotide polymorphisms (SNPs) related to semen production traits in Chinese Holstein bulls. Among them, three SNPs were within or close to the *phosphodiesterase 3A* (*PDE3A*), *membrane associated ring-CH-type finger 1* (*MARCH1*) and *platelet derived growth factor receptor beta* (*PDGFRB*) genes. The present study was designed with the objectives of identifying genetic polymorphism of the *PDE3A*, *PDGFRB* and *MARCH1* genes and their effects on semen production traits in a Holstein bull population.

**Results:**

A total of 20 SNPs were detected and genotyped in 730 bulls. Association analyses using de-regressed estimated breeding values of each semen production trait revealed four statistically significant SNPs for one or more semen production traits (*P* < 0.05): one SNP was located downstream of *PDGFRB* and three SNPs were located in the promoter of *MARCH1*. Interestingly, for *MARCH1*, haplotype-based analysis revealed significant associations of haplotypes with semen volume per ejaculate. Furthermore, high expression of the *MARCH1* gene was observed in sperm cells. One SNP (rs43445726) in the regulatory region of *MARCH1* had a significant effect on gene expression.

**Conclusion:**

Our study demonstrated the significant associations of genetic variants of the *PDGFRB* and *MARCH1* genes with semen production traits. The identified SNPs may serve as genetic markers to optimize breeding programs for semen production traits in Holstein bull populations.

**Electronic supplementary material:**

The online version of this article (doi:10.1186/s12863-017-0527-1) contains supplementary material, which is available to authorized users.

## Background

In livestock breeding, the diagnosis of male fertility is very important because about half of pregnancy failures can be attributed to decreased male fertility or male factor infertility [1]. Sires with subfertility problems lead to larger economic losses than infertile ones because the latter can be detected early while the former require a long period of observation. The significant economic importance of male fertility is also relevant in dairy cattle, especially in the situation in which artificial insemination is widely used. The quality and quantity of semen can be measured by semen production traits, such as semen volume, sperm motility and sperm concentration, as well as observations of abnormal spermatozoa. Semen production traits are complicated, being affected by many nongenetic factors such as age, season, interval between ejaculations and bull handlers, as well as genetic factors [2–4]. Semen volume, sperm concentration and the number of spermatozoa have been estimated to have moderate heritability (0.15–0.30), while sperm motility has been found to be highly heritable (close to 0.6) [4]. Similar results were obtained by Karoui et al., namely, that heritability estimates for semen production traits were moderate (0.16–0.22) [5]. Therefore, genetic improvement of these traits via selection is possible.

Many candidate genes for semen production traits have been revealed using candidate association analysis and genome-wide association study (GWAS). Fortes et al. detected the most significant SNPs in the X chromosome associated with the percentage of progressive motile spermatozoa at 18 months of age and the percentage of normal spermatozoa at 24 months of age [6]. Hering et al. also highlighted several candidate genes associated with sperm concentration, sperm motility and sperm volume in Holstein-Friesian populations [7–9]. In addition, some genes such as *FSHR*, *INHA*, *TNP1*, *TNP2*, *CAPN1* and *SPAG11* have been widely studied as candidate genes for semen production traits of bulls [10–14]. Using Illumina Bovine SNP50 Beadchip (Illumina Inc., San Diego, CA, USA), our previous GWAS detected 19 significant SNPs for five semen production traits in a population of 692 Chinese Holstein bulls [15]. Of those, three SNPs located within or close to the *phosphodiesterase 3A* (*PDE3A*), *platelet derived growth factor receptor beta* (*PDGFRB*) and *membrane associated ring-CH-type finger 1* (*MARCH1*) genes were significantly associated with initial sperm motility (*P* = 3.31E^−05^), sperm volume per ejaculate (*P* = 3.75E^−05^) and sperm volume per ejaculate (*P* = 6.00E^−05^), respectively.

In this study, we aim to investigate genetic variants potentially related to semen production traits in an independent dairy cattle population. We also explore the potential impact of SNP variation in regulatory regions of the above genes on gene expression.

## Methods

### Resource population and analysis of de-regressed EBVs

A total of 730 Chinese Holstein bulls were selected without overlapping with the population of our previous GWAS to construct a single population in this study. The semen production traits included the semen volume per ejaculate [SVPE (ml)], the initial sperm motility [SMOT (%)], the sperm concentration per ejaculate [SCPE (×10^8^/ml)], the number of sperm per ejaculate [NSPE (×10^8^), equal to SVPE multiplied by SCPE] and the number of motile sperm per ejaculate [NMSPE (×10^8^), equal to NSPE multiplied by SMOT]. The breeding values (EBVs) and accuracies of EBVs of selected bulls for five semen production traits were calculated using the AI_REML procedure in the DMU package based on records of 335,005 ejaculate samples from 1450 Chinese Holstein bulls aged 14–144 months. The linear model used was as follows:

$$ {\mathrm{y}}_{i j klmnopq}=\upmu +{F}_i+{H}_{i j}+{A}_k+{S}_l+{T}_m+{I}_n+{\alpha}_o+{PE}_p+{\varepsilon}_{i j klmnopq} $$

where y_*ijklmnopq*_ is the phenotypic value of each trait of bulls; μ is the overall mean; *F*_*i*_ represents the fixed effect of farm; *H*_*ij*_ represents handlers of semen collection, which is nested in the farm effect; *A*_*k*_ represents the fixed effect of age; *S*_*l*_ represents the fixed effect of the season when frozen semen samples were collected; *T*_*m*_ represents the number of collections on one day; *I*_*n*_ represents the interval (in days) between collections; *α*_*o*_ is the random polygenic effect, distributed as N (0, A${\upsigma}_a^2$) with the polygenic relationship matrix A and the additive genetic variance ${\sigma}_a^2$; *PE*_*p*_ is the permanent environment effect; and *ε*_*ijklmnopq*_ is the random residual, distributed as N (0, I${\upsigma}_e^2$) with identity matrix I and residual error variance ${\upsigma}_e^2$.

The EBVs were de-regressed, and the weights were calculated using the method proposed by Garrick et al. (2009) [16]. The descriptive statistics of the de-regressed and original EBVs for five semen production traits in the 730 bulls are listed in Table 1.

**Table 1:** Descriptive statistics of estimated breeding values (EBVs) for the five semen production traits in this study

Traits	No. of bulls	De-regressed EBV		Original EBV		
		Mean	SD	Mean	SD	Mean accuracy
SVPE (ml)	730	0.03	2.04	0.09	0.84	0.65 ± 0.03
SMOT (%)	730	−0.26	6.38	0.21	3.13	0.71 ± 0.02
SCPE (×10^8^/ml)	730	−0.19	3.40	−0.15	1.60	0.70 ± 0.02
NSPE (×10^8^)	730	−0.29	26.68	0.37	13.23	0.71 ± 0.02
NMSPE (×10^8^)	730	−0.06	19.91	0.44	8.65	0.67 ± 0.02

The levels of heritability of SVPE, SMOT, SCPE, NSPE and NMSPE were estimated to be 0.15, 0.12, 0.22, 0.16 and 0.12, respectively. Positive genetic correlations were observed among all traits, and the highest correlation was observed between NSPE and NMSPE (Table 2).

**Table 2:** Heritability and genetic correlations of the five semen production traits

	SVPE (ml)	SMOT (%)	SCPE (×10^8^/ml)	NSPE (×10^8^)	NMSPE (×10^8^)
SVPE (ml)	0.15				
SMOT (%)	0.36	0.12			
SCPE (×10^8^/ml)	0.02	0.18	0.22		
NSPE (×10^8^)	0.70	0.25	0.71	0.16	
NMSPE (×10^8^)	0.74	0.25	0.65	0.99	0.12

Values on the diagonal are the heritability of each trait and values below the diagonal are the genetic correlations between traits

### SNP identification and genotyping

Genomic DNA was isolated from the frozen semen of 730 bulls using a standard phenol-chloroform method. The quality and quantity of extracted genomic DNA were measured with a NanoDrop™ Spectrophotometer (ND-2000c) (Thermo Scientific, Chelmsford, MA, USA) and gel electrophoresis. Then, each DNA sample was diluted to 50 ng/μL and stored at −20 °C for subsequent use. A DNA pool was constructed from 50 randomly selected samples with equal amounts of DNA (50 ng/μL). A total of 83 pairs of primers were designed to amplify entire coding regions, partial introns, and 5′ upstream (3000 bp) and 3′ downstream regions (3000 bp) based on the genomic sequences of the bovine *PDE3A*, *PDGFRB* and *MARCH1* genes (NCBI accession no. AC_000162.1, AC_000164.1 and AC_000163.1). PCR amplifications for pooled DNA were performed in a reaction volume of 20 μL comprising 2 μL of 50 ng/μL DNA, 1 μL of each primer, 10 μL of premix (containing dNTPs and DNA polymerase) (Tiangen, Beijing, China) and 6 μL of ddH_2_O. The amplification procedures were as follows: 10 min at 95 °C for initial denaturing; followed by 35 cycles at 95 °C for 30 s, 60 °C for 30 s and 72 °C for 30 s; and a final extension at 72 °C for 10 min. Amplification products were confirmed by gel electrophoresis on 2% agarose gels and sequenced using ABI3730XL (Applied Biosystems). Furthermore, the identified SNPs were genotyped in 730 Chinese Holstein bulls using a matrix-assisted laser desorption/ionization time of flight mass spectrometry assay (MALDI-TOF-MS; Squenom MassARRAY, Bioyong Technologies Inc., Hong Kong).

### Linkage disequilibrium (LD) analysis and haplotype construction

Hardy–Weinberg equilibrium was tested on each identified SNP using the chi-squared test at a *P*-value cut-off of 0.01. To estimate the extent of LD for the three genes, pairwise LD was measured among the SNPs of each gene based on the criterion of D’ using the software Haploview 4.2 (Broad Institute of MIT and Harvard, Cambridge, MA, USA) [17]. Accordingly, haplotype blocks where SNPs were in high LD (D’ > 0.90) were also determined based on confidence interval methods [18]. A haplotype with a frequency > 5% was treated as a distinguishable haplotype, and those haplotypes with relative frequency < 5% were pooled into a single group. Haplotype blocks within these SNPs were later employed to test their associations with the semen production traits in subsequent analyses.

### Analyses of associations with semen production traits

Pedigree information of the resource population was traced back for three generations to construct the numerator relationship matrix. The associations of SNPs and haplotypes with the five semen production traits were evaluated using the mixed procedure in SAS 9.3 (SAS Institute Inc., Cary, NC). The model was performed as follows:

$$ {\mathrm{y}}_{i jk}=\mu +{G}_i+{\alpha}_j+{e}_{i jk} $$

where y_*ijk*_ is the de-regressed EBVs; *μ* is the overall mean of de-regressed EBVs; *G*_*i*_ is the fixed effect corresponding to the genotype of polymorphisms or haplotypes; *α*_*j*_ is the random familial polygenic effect, distributed as N (0, A${\sigma}_a^2$), with the polygenic relationship matrix A and the additive genetic variance ${\sigma}_a^2$; and *e*_*ijk*_ is the random residual, distributed as N (0, $\mathrm{I}{\upsigma}_e^2$), with identity matrix I and residual error variance ${\upsigma}_e^2$. Values at *P* < 0.05 were considered significant while values at *P* < 0.01 were regarded as highly significant. The differences among the effects of three genotypes on each SNP or haplotype were compared using multiple *t*-test with Bonferroni correction. In addition, the Bonferroni-corrected significance levels of 0.05/3 = 0.0167 and 0.01/3 = 0.0033 were used for comparison of the three genotypes. For the haplotypes, Bonferroni-corrected significance levels of 0.05/N and 0.01/N were used, where N represents the number of formed haplotypes in a block. Moreover, the additive (a), dominance (d) and allele substitution (α) were calculated according to the equation proposed by Falconer & Mackay [19], namely, a = (AA − BB)/2; d = AB – (AA + BB)/2; and α = a + d *(*p* − *q*). Here, AA and BB are the genotype frequencies of the two homozygotes; AB is the heterozygous genotype frequency; and *p* and *q* are the allele frequencies at the corresponding locus.

The percentage of genetic variance accounted for by the significant *i*-th SNP was estimated according to the formula below [20]:

$$ {\% V}_i=100\times \frac{2{p}_i{q}_i{a}_i^2}{\sigma_a^2} $$

where *p*_*i*_ and *q*_*i*_ are the allele frequencies for the significant *i*-th SNP, ${a}_i^2$ is the estimated additive effect of the significant *i*-th SNP on the trait under analysis and ${\sigma}_a^2$ is the additive genetic variance for the trait.

### Gene expression assays of *PDE3A*, *PDGFRB* and *MARCH1* genes

To further confirm the potential functions of the *PDE3A*, *PDGFRB* and *MARCH1* genes, we conducted gene expression analyses of different genotypes. Fresh semen samples were collected from ten fully genotyped bulls.

Fresh semen samples were carefully laid on a monolayer of 40% Percoll. Somatic cell contamination of the sperm cells was removed by centrifugation at room temperature for 20 min at 2000 rpm. After removal of the Percoll solution, sperm pellets were washed twice in 5 mL of warm-up phosphate-buffered saline for 20 min at 2000 rpm. Total RNA extraction of sperm pellets was performed using the standard TRIzol method. The quality and quantity of RNA were measured using an Agilent 2100 Bioanalyzer. Reverse transcription was conducted using a PrimeScript® 1st Strand cDNA Synthesis kit (TaKaRa, Dalian, China), following the manufacturer’s instructions. The primers for *PDE3A*, *PDGFRB*, *MARCH1* and the housekeeping gene *GAPDH* were designed by Primer-Blast on NCBI and synthesized by Beijing Genomics Institute Tech. (Table 3). The reverse-transcription reaction was performed as follows: 10 min at 95 °C for initial denaturing; followed by 35 cycles at 95 °C for 30 s, 60 °C for 30 s and 72 °C for 30 s; and a final extension at 72 °C for 10 min. Amplification products were confirmed by gel electrophoresis on 2% agarose gels to check primer specificity and feasibility. Real-time PCR using SYBR green fluorescence (Roche, Penzberg, Germany) was performed with a volume of 15 μL containing 7.5 μL of SYBR Green Mixture, 2 μL of cDNA template (50 ng/μL), 0.375 μL of each primer (10 μM) and 4.75 μL ddH_2_O. The PCR conditions were as follows: denaturation at 95 °C for 2 min, followed by amplification for 45 cycles of 95 °C for 10 s and 60 °C for 30 s. The last stage used for the dissociation curve was as follows: 95 °C for 15 s, 65 °C for 10 s and 97 °C for 60 s. Quantitative real-time PCR analysis of each gene was performed in triplicate and the relative gene expression was normalized using *GAPDH* by the 2^−ΔΔCt^ method, as described previously [21].

**Table 3:** Primers used to determine the relative expression of the *PDE3A*, *PDGFRB* and *MARCH1* genes as well as *GAPDH*

Gene name	Primer sequence (5′–3′)	Fragment size (bp)	Annealing temperature (°C)
*PDE3A*	F: TCCCCAGGGAACAGCTCAT	141	60
	R: CTGCCAGGAGGTCAGTGATG		
*PDGFRB*	F: AGGCATCAGCAGCAAGGATAC	106	60
	R: CTGTGGTCCCAGCAGAAACA		
*MARCH1*	F: GCGTGGTCTGGTCCTTGTAT	84	60
	R: GCCATTCGAGGACACCGTTA		
*GAPDH*	F: AATGGAAAGGCCATCACCATC	136	60
	R: GTGGTTCACGCCCATCACA		

To further detect the effect of variants of significantly associated genes, the mRNA expression of sperm cells with different genotypes at sites of functionally important mutations was analyzed. The results of mRNA expression were analyzed by the GLM procedure in SAS 9.3 software.

## Results

### Identification of SNPs in *PDE3A, PDGFRB* and *MARCH1*

Sequence analysis revealed that a total of 20 SNPs were detected using the pooled DNA of 50 bulls. Of those, eight SNPs were located in *PDE3A*, being distributed in exons (*n* = 2), introns (*n* = 3) and the 3′ untranslated region (*n* = 3). In addition, five SNPs were located in *MARCH1*, being distributed in the promoter (*n* = 3), an exon (*n* = 1) and an intron (*n* = 1). Furthermore, seven SNPs were located in *PDGFRB*, being distributed in exons (*n* = 2), introns (*n* = 2) and downstream of the gene (*n* = 3). The identified SNPs were then subjected to genotyping in 730 bulls. However, not all individuals were successfully genotyped at all SNPs. The number of remaining individuals as well as genotype frequencies, allele frequencies, primers for amplification and results of the chi-squared tests for each SNP are shown in Additional file 1: Table S1. Only one SNP, rs109116577, was a missense mutation, causing an amino acid change of Pro/Ser in the PDE3A protein. Three SNPs (rs456212302, rs42393923 and rs378918630) that were not in Hardy–Weinberg equilibrium were excluded from subsequent analysis (*P* < 0.01).

### Association between SNPs and semen production traits in Chinese Holstein bulls

Association studies showed that four statistically significant SNPs associated with at least one semen production trait. The estimated effects of the four significant SNPs on semen production traits are shown in Table 4.

**Table 4:** Associations of the four significant SNPs in *PDGFRB* and *MARCH1* with semen production traits in Chinese Holstein bulls (LSM ± SE)

Genes	SNPs	Genotypes (no.)	SVPE (ml)	SMOT (%)	SCPE (×10^8^/ml)	NSPE (×10^8^)	NMSPE (×10^8^)
*PDGFRB*	rs110305039	GG (269)	0.243 ± 0.159^A^	0.676 ± 0.739	0.054 ± 0.256	3.030 ± 2.440^a^	2.400 ± 1.811^a^
		GT (359)	0.028 ± 0.138^A^	0.379 ± 0.962	−0.238 ± 0.222	−0.520 ± 2.112^ab^	−0.229 ± 1.568^ab^
		TT (81)	−0.830 ± 0.289^B^	0.774 ± 0.871	−0.627 ± 0.467	−11.124 ± 4.447^b^	−8.184 ± 3.301^b^
		*p*-value	0.0052**	0.6581	0.4034	0.0206*	0.0195*
		%Var	11.67			7.92	9.97
*MARCH1*	rs211260176	CC (131)	−0.342 ± 0.228^a^	0.845 ± 1.019	−0.184 ± 0.367	−3.837 ± 3.496	−2.844 ± 2.595
		CT (349)	−0.060 ± 0.140^ab^	0.678 ± 0.625	−0.296 ± 0.225	−1.493 ± 2.142	−0.847 ± 1.590
		TT (231)	0.354 ± 0.171^b^	0.606 ± 0.766	−0.029 ± 0.276	3.301 ± 2.628	2.519 ± 1.951
		*p*-value	0.0361*	0.3588	0.7537	0.2014	0.2083
		%Var	5.19				
	rs208093284	CC (126)	−0.337 ± 0.232^a^	−0.881 ± 1.039	−0.129 ± 0.374	−3.481 ± 3.565	−2.576 ± 2.646
		CT (311)	−0.124 ± 0.148^ab^	−0.857 ± 0.662	−0.323 ± 0.238	−2.211 ± 2.269	−1.368 ± 1.684
		TT (270)	0.342 ± 0.158^b^	0.623 ± 0.709	−0.133 ± 0.256	2.619 ± 2.434	2.041 ± 1.806
		*p*-value	0.0246*	0.2578	0.8348	0.2342	0.2445
		%Var	4.84				
	rs43445726	CC (27)	−1.039 ± 0.501^A^	−2.929 ± 2.245	−0.446 ± 0.808	−11.113 ± 7.700^ab^	−8.185 ± 5.716
		CT (225)	−0.499 ± 0.175^A^	−1.344 ± 0.785	−0.316 ± 0.283	−5.583 ± 2.691^a^	−4.037 ± 1.998
		TT (471)	0.296 ± 0.122^B^	0.287 ± 0.546	−0.123 ± 0.197	2.384 ± 1.872^b^	1.929 ± 1.389
		*p*-value	0.0001**	0.1179	0.8128	0.0203*	0.0189*
		%Var	12.22			4.87	6.16

*P*-value is the significance level from analyses of the association of SNPs with semen production traits. **: *P* < 0.01; *: *P* < 0.05. Different superscript letters (lower-case letters: *P* < 0.05; upper-case letters: *P* < 0.01; Bonferroni-adjusted value after multiple testing) refer to significant differences among the genotypes. %Var indicates the percentage of genetic variance explained by the significant SNPs for traits

One SNP (rs110305039) located downstream of *PDGFRB* was significantly related to SVPE (*P* = 0.0052), NSPE (*P* = 0.0206) and NMSPE (*P* = 0.0195). In particular, SNP rs110305039 had been revealed as an SNP significantly associated with sperm volume in our previous GWAS [15].

Three SNPs (rs211260176, rs208093284 and rs43445726) located in the promoter of *MARCH1* were significantly associated with SVPE (*P* = 0.0246); SVPE (*P* = 0.0341); and all three of SVPE (*P* = 0.0001), NSPE (*P* = 0.0203) and NMSPE (*P* = 0.0189), respectively. In addition, the results showed that homozygous genotypes of all the significant SNPs were dominant for semen production traits. The dominant, additive and allele substitution effects of the significant SNPs on the target semen production traits are presented in Table 5.

**Table 5:** The dominant (d), additive (a) and allele substitution (α) effects of the significant SNPs in *PDGFRB* and *MARCH1* genes on the five semen production traits

Gene	SNPs	Genetic effect	SVPE (ml)	SMOT (%)	SCPE (×10^8^/ml)	NSPE (×10^8^)	NMSPE (×10^8^)
*PDGFRB*	rs110305039	a	0.536**	0.676	0.340	7.077**	5.292**
		d	0.322	0.379	0.048	3.527	2.663
		α	0.619**	0.774	0.353	7.986**	5.978**
*MARCH1*	rs211260176	a	−0.340*	0.752	0.002	−3.050	−2.308
		d	−0.126	0.728	−0.192	−1.780	−1.100
		α	−0.365*	−0.898	−0.037	−3.406	−2.528
	rs208093284	a	−0.348*	−0.726	−0.078	−3.569	−2.682
		d	−0.066	−0.559	−0.190	−1.225	−0.685
		α	−0.357*	−0.804	−0.105	−3.740	2.778
	rs43445726	a	−0.667**	−1.608	−0.162	−6.748	−5.057
		d	−0.127	−0.023	−0.031	−1.219	−0.909
		α	−0.744**	−1.622	−0.180	−7.479	−5.603

A indicates additive effect; d indicates dominant effect; α indicates allele substitution effect; a single asterisk (*) means that the additive, dominance or allele substitution effect of the locus is significant (*P* < 0.05), and double asterisks (**) mean that the additive, dominance or allele substitution effect of the locus is extremely significant (*P* < 0.01)

### LD among identified SNPs and haplotype association results

**Fig. 1:**
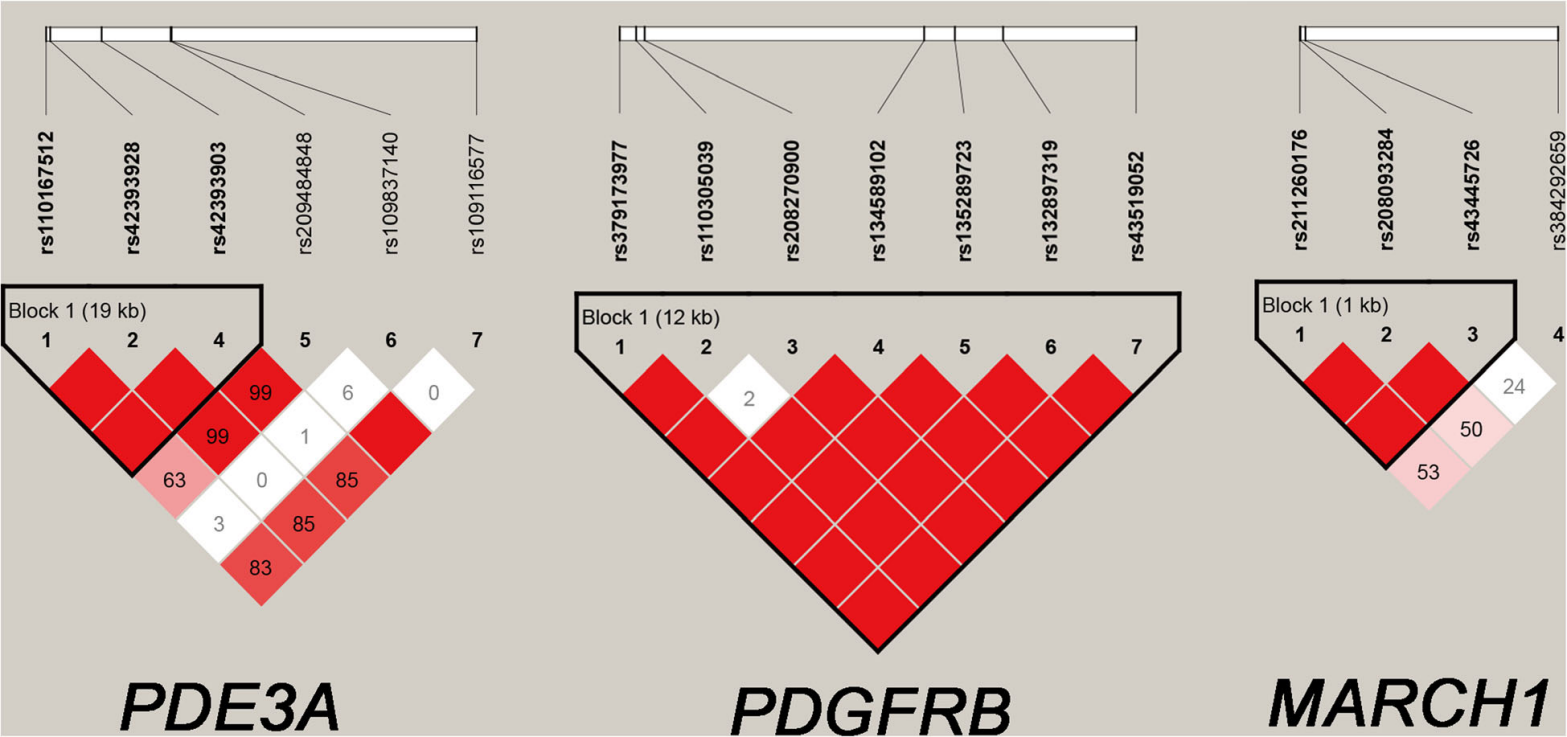
Linkage disequilibrium analyses revealed three blocks for the identified SNPs in the *PDE3A*, *MARCH1* and *PDGFRB* genes. The values in boxes are pairwise SNP correlations (D’), while bright red boxes without numbers indicate complete LD (D’ = 1). The blocks indicate haplotype blocks and the text above the horizontal numbers is the SNP names

Pairwise D’ measures between genotyped SNPs of the three genes were investigated and the inferred haplotype blocks are shown in Fig. 1. For *PDE3A*, one block consisting of three SNPs (rs110167512, rs42393928 and rs42393903) was inferred and three haplotypes were formed in the studied population. Association analysis revealed that haplotypes of *PDE3A* did not reach significance for five semen production traits. For *PDGFRB*, four haplotypes in one block with seven SNPs did not significantly associate with semen production traits. For *MARCH1*, three SNPs (rs211260176, rs208093284 and rs43445726) constituted a block in the studied population. The main haplotypes of TTT (H1), CCT (H2) and CCC (H3) accounted for frequencies of 57.2%, 20.7% and 19.5% of the total, respectively. Haplotype association study of *MARCH1* demonstrated a significant association with SVPE (*P* = 0.0013) (Table 6).

**Table 6:** Haplotype-based association analyses with semen production traits in Chinese Holstein bulls (LSM ± SE)

Gene	Haplotypes (no.)	SVPE (ml)	SMOT (%)	SCPE (×10^8^/ml)	NSPE (×10^8^)	NMSPE (×10^8^)
*PDE3A*	H1H1	0.187 ± 0.150	0.807 ± 0.673	−0.240 ± 0.242	0.226 ± 2.307	0.360 ± 1.711
	H1H2	−0.191 ± 0.157	−1.106 ± 0.705	−0.384 ± 0.253	−3.121 ± 2.417	−2.108 ± 1.794
	H1H3	0.598 ± 0.412	−1.913 ± 1.845	0.589 ± 0.664	11.385 ± 6.326	8.593 ± 4.696
	H2H2	−0.222 ± 0.286	−1.125 ± 1.281	0.137 ± 0.461	−0.386 ± 4.394	−0.415 ± 3.261
	*P*-value	0.1266	0.1591	0.4758	0.1878	0.1924
*PDGFRB*	H1H1	0.110 ± 0.316	−0.438 ± 1.415	−0.151 ± 0.509	0.898 ± 4.852	1.013 ± 3.602
	H1H2	0.302 ± 0.223	0.057 ± 1.000	−0.032 ± 0.360	2.976 ± 3.431	2.508 ± 2.547
	H1H3	0.089 ± 0.269	0.475 ± 1.203	−0.349 ± 0.433	−1.437 ± 4.127	−0.694 ± 3.064
	H1H4	0.149 ± 0.293	0.288 ± 1.313	−0.546 ± 0.473	−1.196 ± 4.502	−0.673 ± 3.342
	H2H2	0.237 ± 0.334	0.520 ± 1.494	0.333 ± 0.538	4.645 ± 5.123	3.095 ± 3.802
	H2H3	−0.042 ± 0.283	−1.458 ± 1.265	0.191 ± 0.456	2.214 ± 4.340	1.528 ± 3.221
	H2H4	−0.119 ± 0.286	−0.541 ± 1.281	−0.073 ± 0.461	−1.039 ± 4.392	−0.638 ± 3.260
	H3H3	−0.821 ± 0.598	−1.814 ± 2.676	−1.141 ± 0.964	−13.215 ± 9.179	−9.594 ± 6.814
	H3H4	−0.827 ± 0.423	1.172 ± 1.892	−0.400 ± 0.682	−10.118 ± 6.491	−7.527 ± 4.818
	H4H4	−0.612 ± 0.568	1.703 ± 2.546	−0.470 ± 0.917	−7.925 ± 8.731	−5.668 ± 6.481
	*P*-value	0.3630	0.9750	0.9280	0.5946	0.6112
*MARCH1*	H1H1 (231)	0.364 ± 0.171^a^	0.622 ± 0.768	−0.033 ± 0.276	3.347 ± 2.633	2.557 ± 1.954
	H1H2 (149)	0.248 ± 0.213^ab^	−0.421 ± 0.956	−0.250 ± 0.344	1.502 ± 3.278	1.519 ± 2.433
	H1H3 (156)	−0.456 ± 0.210^b^	−1.372 ± 0.931	−0.346 ± 0.335	−5.223 ± 3.193	−3.711 ± 2.370
	H2H2 (38)	0.519 ± 0.423^ab^	0.987 ± 1.892	0.469 ± 0.682	7.373 ± 6.491	5.449 ± 4.818
	H2H3 (58)	−0.664 ± 0.342^ab^	−1.369 ± 1.532	−0.376 ± 0.552	−7.913 ± 5.254	−5.872 ± 3.900
	H3H3 (27)	−1.030 ± 0.501^ab^	2.910 ± 2.245	−0.443 ± 0.808	−11.030 ± 7.699	−8.129 ± 5.715
	*p*-value	0.0013**	0.4109	0.8877	0.0760	0.0740

*P*-value is the significance level from analyses of the association of haplotypes with semen production traits. **: *P* < 0.01. Superscript letters (*P* < 0.05; Bonferroni-adjusted value after multiple testing) refer to a significant difference among the genotypes

### Functional prediction of the allele-dependent transcription factor binding sites

**Fig. 2:**
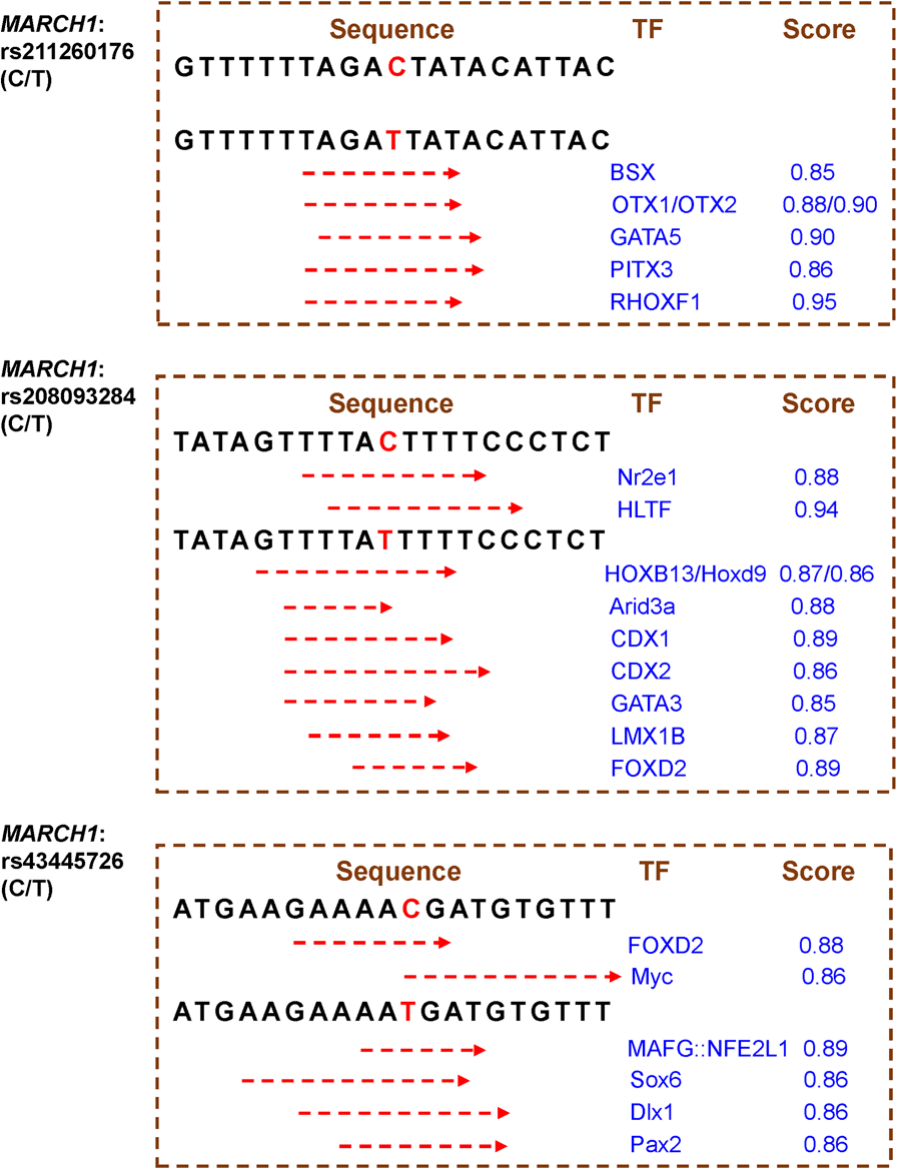
The changed transcription factor binding sites due to allele substitution of significant SNPs in the regulatory region of *MARCH1*. The significant SNPs in sequences are highlighted in red. The red dotted lines indicate the predicted transcription factor binding sites

The mutations in the regulatory regions of a gene can affect the transcription rate by changing the transcription factor binding sites [22]. Therefore, three significant SNPs located in the promoter of *MARCH1* may be involved in altered transcription factor binding sites and may subsequently lead to gene expression differences. Sequences including the significant SNPs (21 bp) were subjected to a comparison with the reference transcription factor binding sites in the JASPAR CORE Vertebrata database (http://jaspar.genereg.net/cgi-bin/jaspar_db.pl), using a relative profile score threshold of 85%. As a result, the three regulatory SNPs were predicted to create some new transcription binding sites via the substitution of C to T. The details of this are shown in Fig. 2.

### Expression regulation of the mutations in the *MARCH1* Gene

**Fig. 3:**
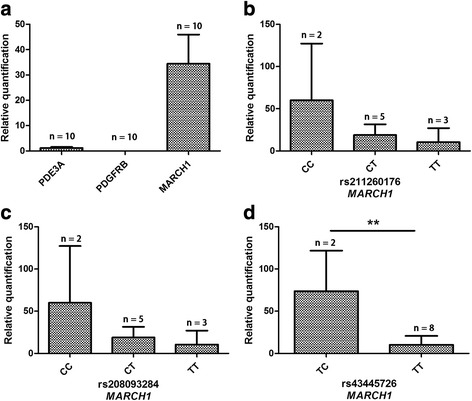
Normalized mRNA expression of *PDE3A*, *PDGFRB* and *MARCH1* genes and associations between significant SNPs and the expression level of *MARCH1* in semen samples. **a** Normalized mRNA expression of *PDE3A*, *PDGFRB* and *MARCH1* genes in semen samples. **b** SNP of *MARCH1*, rs211260176: no significant difference of expression levels was detected among CC (*n* = 2), CT (*n* = 5) and TT (*n* = 3), *P*-value: 0.1918. **c** SNP of *MARCH1*, rs208093284: no significant difference of expression levels was detected among CC (*n* = 2), CT (*n* = 5) and TT (*n* = 3), *P*-value: 0.1918. **d** SNP of *MARCH1*, rs43445726: extremely significant difference of expression was detected between TC (*n* = 2) and TT (*n* = 8), *P*-value: 0.0035

The mRNA expression of the three genes was determined by quantitative real-time PCR and normalized using internal *GAPDH* expression in sperm cells. *MARCH1* had a higher expression than the other two genes in sperm cells (Fig. 3a). To investigate the potential regulatory role of SNPs in the 5′ regulatory region, association analyses between different genotypes and *MARCH1* expression level were conducted. We found that the two genotypes (CC was not observed) of rs43445726 were associated with a significant difference in gene expression of *MARCH1* (*P* = 0.0035) (Fig. 3d). Similarly, another two SNPs of *MARCH1* showed a tendency for genotype-specific gene expression (both *P* = 0.1918) (Fig. 3b, c).

## Discussion

In support of our previous GWAS, we provide further evidence for the significant genetic effects of the *PDGFRB* and *MARCH1* genes on semen production traits in another population of Chinese Holstein bulls.

The PDE3A enzyme, mainly located in the post-acrosomal segment of the sperm head [23], has been reported to exhibit activity in catalyzing cAMP into 5′AMP in spermatozoa. cAMP is an important secondary messenger in the control of sperm functions, encompassing activation of motility, the acrosome reaction, hyperpolarization of sperm plasma membrane and ATP analysis [24]. In normal spermatozoa, PDE3A activity is inhibited by cGMP, maintaining a high cAMP level [25]. *PDE3A* is only 0.1 Mb away from another SNP found to be significantly associated with sperm motility in our previous GWAS. It is also close to an SNP (1.8 Mb away) shown to be significantly associated with sperm motility in a Polish Holstein bull population [8], and about 2.3 Mb away from an SNP significant for sire conception rate in another GWAS [26]. However, we did not observe significant associations between mutations in *PDE3A* and five semen production traits. We suspected that *PDE3A* might have genetic effects on semen production traits, but the causal mutation has not been detected in the studied population. Further studies should be conducted to confirm the genetic effects of *PDE3A* on semen production traits.

One SNP situated downstream of *PDGFRB* was found to have significant associations with SVPE, NSPE and NMSPE, which had already been declared to be significantly associated with SVPE in our previous GWAS. The mRNA of *PDGFRB* has been detected in gonocytes [27], Leydig and Sertoli cells, but not round spermatids or primary spermatocytes [28]. Similarly, we also did not detect the expression of *PDGFRB* in sperm cells. Gonocytes, the precursors of spermatogonial stem cells, are located in the center of the seminiferous tubules. At a defined species-specific period of time (cattle: pre-puberty), their proliferation and migration to the basement membrane give rise to spermatogonial stem cells, which maintain spermatogenesis in the mature testis [29]. The PDGFRB protein has been reported to play a leading role during the proliferation and migration of gonocytes [27]. The inhibition of PDGFRB tyrosine kinase activity was shown to reduce testis size, delay the initiation of spermatogenesis and thus provoke a drastic reduction of epididymal sperm count [27].

MARCH proteins are ubiquitin ligases and target glycoproteins for lysosomal destruction via ubiquitination of the cytoplasmic tail. Unlike the above two candidate genes, the functions of *MARCH1* are seldom analyzed in relation to spermatogenesis. However, previous studies identified that three MARCH family members, *MARCH-XI*, *MARCH10* and *MARCH7*, are highly expressed in developing spermatids [30–32]. The MARCH-XI protein is postulated to be a ubiquitin ligase that mediates transmembrane glycoproteins in the trans-Golgi network and multivesicular body transport pathway, which is associated with acrosomal formation in developing spermatids [30]. *MARCH10* is abundantly expressed in elongated spermatids. Furthermore, immunohistochemical analysis of MARCH10 proteins revealed that they are predominantly located in the cytoplasmic lobes and the principal piece of the flagella. It is supposed that MARCH10 proteins are synthesized in the cytoplasm and then transported to the developing flagella [31]. Similarly, MARCH7 proteins that are localized to the acroplaxome and flagella mediate K48-linked ubiquitination in the acrosome/acroplaxome region and may be related to the regulation of head shaping and flagellar formation in developing spermatids [32]. In our study, the high expression of *MARCH1* in spermatozoa and significant effects of *MARCH1* on SVPE, SNPE and SNMPE support the asumption that *MARCH1* functions in spermatogenesis, as *MARCH-XI*, *MARCH10* and *MARCH7* do.

In the present study, alleles involving a substitution of C to T in the regulatory region of *MARCH1* were predicted to add a series of transcription factor binding sites. Specifically, one of the added transcription factors, RHOXF1, is encoded by an X-linked reproductive homeobox gene and has been observed to be specifically expressed in testis, especially in pachytene spermatocytes and round spermatids [33]. RHOXF1 critically upregulates many genes in male reproduction and may also modulate the transcription of *MARCH1* [34]. Furthermore, one of the significant SNPs, rs43445726, resulted in a marked difference in expression level, with expression associated with the CT genotype being seven times that for the TT genotype. As for the phenotypes, CT was associated with lower sperm volume and sperm number than TT, which reflected the negative effect of *MARCH1* on SVPE, NSPE and NMSPE. Furthermore, it was predicted that the SNP rs43445726 explained 12.22% of the genetic variance of SVPE, implying significant genetic effects of this mutation. However, the detected effects may be limited to the specific population studied here, so further analysis should be conducted to reveal the function of *MARCH1* in spermatogenesis and verify the functional implications of its mutations.

## Conclusion

Our findings demonstrated that *PDGFRB* and *MARCH1* were significantly associated with semen production traits and presented the high expression of *MARCH1* in mature sperm, which were consistent with previous GWAS and functional analyses. Our results not only provide new insight into the functions of the *PDGFRB* and *MARCH1* genes, but also contribute useful information for marker-assisted selection or genome selection strategies of genetic improvement programs for semen production traits.

## Additional file

Additional file 1: Table S1. Genotype frequencies, allele frequencies, results of the chi-squared tests of identified SNPs in 730 Chinese Holstein bulls and their corresponding primers for the SNP detection of *PDE3A*, *MARCH1* and *PDGFRB* genes. (DOCX 30 kb)

## Abbreviations

EBV: Evaluated breeding value
GWAS: Genome-wide association study
LD: Linkage disequilibrium
MARCH1: Membrane associated ring-CH-type finger 1
MARCH10: Membrane associated ring-CH-type finger 10
MARCH7: Membrane associated ring-CH-type finger 7
MARCH-XI: Membrane associated ring-CH-type finger XI
NMSPE: Number of motile sperm per ejaculate
NSPE: Number of sperm per ejaculate
PDE3A: Phosphodiesterase 3A
PDGFRB: Platelet derived growth factor receptor beta
RHOXF1: Rhox homeobox family, member 1
SCPE: Sperm concentration per ejaculate;
SMOT: Initial sperm motility
SNP: Single-nucleotide polymorphism
SVPE: Semen volume per ejaculate
