# Supplementary material for: Genetic effects of PDGFRB and MARCH1 identified in GWAS revealing strong associations with semen production traits in Chinese Holstein bulls
Source: BMC Genet. 2017 Jul 3;18:63. doi: 10.1186/s12863-017-0527-1 (PMC5496367; doi:10.1186/s12863-017-0527-1)
Supplement: Additional file 1: Table S1. — Genotype frequencies, allele frequencies, results of the chi-squared tests of identified SNPs in 730 Chinese Holstein bulls and their corresponding primers for the SNP detection of PDE3A, MARCH1 and PDGFRB genes. (DOCX 30 kb) [file 12863_2017_527_MOESM1_ESM.docx]

**Table S1.** Genotype frequencies, allele frequencies, results of the chi-squared tests of identified SNPs in 730 Chinese Holstein bulls and their corresponding primers for the SNP detection of *PDE3A*, *MARCH1* and *PDGFRB* genes.

| SNPs | Location | Genotype (no.) | Genotypic frequency (%) | Alleles | Allele frequency (%) | Primers | χ^2^ (*P*-value) |
| --- | --- | --- | --- | --- | --- | --- | --- |
| rs456212302 | *PDE3A*  (exon 1) | CC (1) | 0.00 | C | 0.00 | F: TCTGGATGGGCTTGTACCTC  R: tgcaggtccttacCTGTTCC | 730.00 (0.0007) |
|  |  | CG (0) | 0.00 |  |  |  |  |
|  |  | GG (712) | 1.00 | G | 1.00 |  |  |
| rs109116577 | *PDE3A*  (exon 2) | AA (81) | 0.11 | A | 0.35 | F: tggacagagtagtctggaaagc  R: aacaaatggcatgactgctg | 1.51 (0.2545) |
|  |  | AG (337) | 0.48 |  |  |  |  |
|  |  | GG (293) | 0.41 | G | 0.65 |  |  |
| rs109837140 | *PDE3A*  (intron 6) | CC (134) | 0.19 | C | 0.43 | F: ccaaagggaaggctaagagc  R: acaaagctatgtgggctggt | 0.00 (1.0000) |
|  |  | CT (347) | 0.49 |  |  |  |  |
|  |  | TT (226) | 0.32 | T | 0.57 |  |  |
| rs209484848 | *PDE3A*  (intron 5) | AA (14) | 0.02 | A | 0.14 | F: ccaaagggaaggctaagagc  R: acaaagctatgtgggctggt | 0.00 (1.0000) |
|  |  | AG (179) | 0.25 |  |  |  |  |
|  |  | GG (515) | 0.73 | G | 0.86 |  |  |
| rs42393903 | *PDE3A*  (intron 13) | CC (304) | 0.43 | C | 0.66 | F: gggaaaggaagagtggcttt  R: ttggtgcctgctatacgtca | 0.12 (0.7421) |
|  |  | CT (318) | 0.45 |  |  |  |  |
|  |  | TT (86) | 0.12 | T | 0.34 |  |  |
| rs42393923 | *PDE3A*  (3’UTR) | AA (45) | 0.06 | A | 0.12 | F: CTGAGGGATGAAAGCTCCAG  R: TCCCAGGTCTTCTCTGGCTA | 154.21 (0.0000) |
|  |  | AG (84) | 0.12 |  |  |  |  |
|  |  | GG (586) | 0.82 | G | 0.88 |  |  |
| rs42393928 | *PDE3A*  (3’UTR) | CC (300) | 0.43 | C | 0.65 | F: TCCTTGGTGAAAACACCACTT  R: tcatcatttgccagttgttca | 0.02 (0.8696) |
|  |  | CG (320) | 0.45 |  |  |  |  |
|  |  | GG (85) | 0.12 | G | 0.35 |  |  |
| rs110167512 | *PDE3A*  (Downstream) | AA (85) | 0.12 | A | 0.31 | F: AAATTCATGAGACAGGGGAGAA  R: TTTCAGGTAGGCTCTGGCATA | 6.95 (0.0100) |
|  |  | AG (277) | 0.39 |  |  |  |  |
|  |  | GG (346) | 0.49 | G | 0.69 |  |  |
| rs211260176 | *MARCH1*  (Promoter) | CC (126) | 0.18 | C | 0.40 | F: ATTTTGTCCCTCCCCTGTTC  R: CAACCTACGCAACTCAGCAA | 4.47 (0.0364) |
|  |  | CT (311) | 0.44 |  |  |  |  |
|  |  | TT (270) | 0.38 | T | 0.60 |  |  |
| rs208093284 | *MARCH1*  (Promoter) | CC (131) | 0.18 | C | 0.43 | F: ATTTTGTCCCTCCCCTGTTC  R: CAACCTACGCAACTCAGCAA | 0.01 (1.0000) |
|  |  | CT (349) | 0.49 |  |  |  |  |
|  |  | TT (231) | 0.32 | T | 0.57 |  |  |
| rs43445726 | *MARCH1*  (Promoter) | CC (27) | 0.04 | C | 0.19 | F: AGCAAGGAGCAGCACTGATT  R: CACTTTAAGGCCCCATGAGA | 0.01 (1.0000) |
|  |  | CT (221) | 0.31 |  |  |  |  |
|  |  | TT (456) | 0.65 | T | 0.81 |  |  |
| rs384292659 | *MARCH1*  (Exon 2 ) | CC (456) | 0.64 | C | 0.79 | F: cctaggatgctcaaggagga  R: gaagcctgttttgccagttc | 5.25 (0.0247) |
|  |  | CT (216) | 0.30 |  |  |  |  |
|  |  | TT (42) | 0.06 | T | 0.21 |  |  |
| rs378918630 | *MARCH1*  (Intron 3) | GG (678) | 0.95 | G | 0.96 | F: taggcctgggatacaaatgg  R: acaggtgctctgggaaacag | 655.32 (0.0000) |
|  |  | GT (3) | 0.00 |  |  |  |  |
|  |  | TT (28) | 0.04 | T | 0.04 |  |  |
| rs43519052 | *PDGFRB*  (Intron 10) | AA (324) | 0.46 | A | 0.68 | F: ggacgggcagagaggtttt  R: ctagaggatggcagaggtcc | 0.34 (0.6077) |
|  |  | AG (312) | 0.44 |  |  |  |  |
|  |  | GG (70) | 0.10 | G | 0.32 |  |  |
| rs132897319 | *PDGFRB*  (Exon 15) | AA (328) | 0.47 | A | 0.69 | F:cacatgcagcctgtcgttag  R: cctccctgcggtacCTAGG | 0.25 (0.2919) |
|  |  | AG (309) | 0.44 |  |  |  |  |
|  |  | GG (61) | 0.09 | G | 0.31 |  |  |
| rs135289723 | *PDGFRB*  (Exon 16) | AA (63) | 0.09 | A | 0.31 | F: tactgcctctgtgatgctcc  R: ctgggtggaggaaagggttt | 1.50 (0.2606) |
|  |  | AG (318) | 0.45 |  |  |  |  |
|  |  | GG (333) | 0.47 | G | 0.69 |  |  |
| rs134589102 | *PDGFRB*  (Intron 17) | AA (332) | 0.47 | A | 0.69 | F: ggcgagactcaagatgtcct  R: gagggatgctgtatgggtca | 1.50 (0.2606) |
|  |  | AG (318) | 0.45 |  |  |  |  |
|  |  | GG (63) | 0.09 | G | 0.31 |  |  |
| rs208270900 | *PDGFRB*  (Downstream) | GG (178) | 0.25 | G | 0.50 | F: CCTGGTGCTTCTCACTCACA  R: ACAGCCATGAGGGATGAGTC | 0.23 (0.6571) |
|  |  | GT (362) | 0.51 |  |  |  |  |
|  |  | TT (171) | 0.24 | T | 0.50 |  |  |
| rs110305039 | *PDGFRB*  (Downstream) | GG (269) | 0.38 | G | 0.63 | F: CCTGGTGCTTCTCACTCACA  R: ACAGCCATGAGGGATGAGTC | 4.98 (0.0317) |
|  |  | GT (359) | 0.51 |  |  |  |  |
|  |  | TT (81) | 0.11 | T | 0.37 |  |  |
| rs379173977 | *PDGFRB*  (Downstream) | AA (20) | 0.03 | A | 0.19 | F: CCTCAGGCTTGCTCCTTATG  R: CTCATCGTCTGGGAGGAAAA | 1.59 (0.2690) |
|  |  | AG (224) | 0.32 |  |  |  |  |
|  |  | GG (464) | 0.66 | G | 0.81 |  |  |
